# Delivery Challenges for Fluoride, Chlorhexidine and Xylitol

**DOI:** 10.1186/1472-6831-6-S1-S8

**Published:** 2006-06-15

**Authors:** John DB Featherstone

**Affiliations:** 1 Department of Preventive and Restorative Dental Sciences, University of California at San Francisco, San Francisco, CA, USA

## Abstract

The progression or reversal of dental caries is determined by the balance between pathological and protective factors. It is well established that a) fluoride inhibits demineralization and enhances remineralization, b) chlorhexidine reduces the cariogenic bacterial challenge, and c) xylitol is non-cariogenic and has antibacterial properties. The challenge that we face is how best to deliver these anti-caries entities at true therapeutic levels, over time, to favorably tip the caries balance. High caries risk people, including children with Early Childhood Caries (ECC), are a special challenge, since high cariogenic bacterial activity can override fluoride therapy. Current fluoride and chlorhexidine varnishes deliver all their activity within about 24 hours. Early studies with experimental slow release fluoride devices retained elevated levels of fluoride for months in a therapeutic range but have not been pursued. Preventive dentistry has largely ignored the benefits of reducing the bacterial challenge, partially due to primitive and inadequate delivery systems. For example, Chlorhexidine applied as a rinse partially reduces some bacteria but not others that are hiding within the biofilm. Better antibacterials and better delivery systems are needed. Xylitol delivered by gum or lozenge appears to be effective clinically in reducing cariogenic bacteria and caries levels, but novel systems that deliver therapeutic amounts when needed would be a major advance, especially for young children. Reducing the cariogenic bacterial challenge and enhancing the effect of fluoride by the use of new sustained-delivery systems would have a major effect on dealing with caries as a disease.

## Introduction

### The Caries Balance

**Figure 1:**
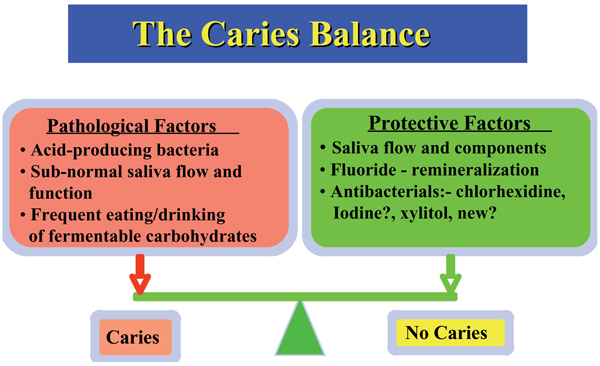
Schematic diagram of the "Caries Balance" (adapted from Featherstone, 2003)[2].

Progression, inhibition, or reversal of dental caries can be determined by considering the "caries balance." This concept was proposed by Featherstone [1] and has subsequently been simplified as the balance between three pathological factors and three protective factors, as illustrated in Figure 1.

Although numerous other factors can be added to either side, these six have been found to be the primary determinants of whether the caries process is in balance, progressing, or reversing [3,4]. This article specifically addresses two protective factors, namely fluoride and extrinsic antibacterials. Although chlorhexidine and xylitol are specifically discussed, the principles apply equally well to any other antibacterial that has an effect on the cariogenic bacteria. For caries prevention or reversal, it is necessary to effectively increase the effect of one or more protective factors or to decrease the effect of one or more pathological factors.

The primary purpose of this article is to discuss the challenges of delivering fluoride, chlorhexidine, and xylitol more effectively. The questions to be answered are:

1) What are the effective therapeutic levels of each of these components?

2) How best can each be delivered to the tooth surface over time?

3) Can new technology deliver known therapeutic agents more effectively and with fewer compliance issues than current methods?

### Fluoride

It is well known that fluoride is a major anticaries agent. The primary mode of action for adults as well as children is through its topical action in the mouth as it reaches the surface of the tooth, the plaque, and the subsurface lesions [5]. Fluoride inhibits demineralization, enhances remineralization, and can inhibit the cariogenic bacteria. For this discussion I will address primarily the role of fluoride in remineralization. Fluoride enhances remineralization of partially dissolved enamel or dentin crystals by combining with calcium and phosphate that comes primarily from saliva. Remineralization is the natural repair process for the non-cavitated carious lesion. Fluoride speeds up remineralization and forms a new fluorapatite-like veneer on the remineralized crystal remnants inside the carious lesion. The crystal solubility is markedly reduced [6].

The level of fluoride needed to enhance remineralization is considerably lower than that needed for the inhibition of demineralization or for an antibacterial effect. Over many years our laboratory has used a pH cycling model to predict likely clinical outcomes. The model was originally developed to mimic the early lesions formed around orthodontic brackets in human mouths [7]. Several variations of the model have been developed, and pH cycling has been shown to be a good indicator of fluoride effects in the mouth [8]. The model consists of alternating periods of demineralization and remineralization in the laboratory over a three-week period. Fifteen years ago we studied the effect of low concentrations of fluoride in the mineralizing solution [8,9]. We studied levels ranging from no added fluoride to 0.5 ppm F to elucidate likely effects of fluoride at these levels in saliva. We found a linear relationship between the logarithm of the fluoride level and the net amount of remineralization. There was a rapid rise in effect as the fluoride concentration increased from 0.03 ppm to 0.1 ppm F. Usual levels in saliva are 0.03 ppm F or less, dependent on the use of fluoride products and fluoride in the drinking water [10]. We predicted that fluoride in saliva at 0.1 ppm would give almost complete protection against caries progression. Of course a simple number such as this does not apply to all situations and is very dependent on the strength of the acid challenge coming form the bacterial fermentation of carbohydrates (see figure 1) and the intensity of the swinging of the caries balance. However, to aim at a continual level of 0.1 ppm F in saliva as a baseline value instead of values of 0.03 ppm or less appears to be sound.

In a recently reported six-year clinical study in children who started caries free at age 6 or 7 years, we found a strong protective effect for salivary fluoride values greater than 0.08 ppm F [11]. Most recently a study by Toumba and Curzon [12] reported a 70% reduction in caries versus control for high risk children who wore a fluoride-releasing glass device in their mouths. The mean salivary F levels were 0.11 ppm in the test group versus 0.03 ppm F in the control group over a two-year period. Baseline fluoride levels in saliva from dentifrice or mouthrinse are generally in the 0.02 – 0.04 ppm F level, which is adequate for low or medium caries challenge individuals, but not for high caries challenge. Even for currently marketed fluoride varnish, these levels are returned to within 24 hours after a varnish application [13].

In earlier studies funded by NIH, various polymer-based slow-release fluoride devices were tested in a variety of retention mechanisms (Billings and co-workers, unpublished data). Again it was illustrated in these in vivo studies that fluoride could be released constantly to give salivary F levels of around 0.1 ppm. These studies have never led to satisfactory commercially viable devices, although the principle was proven.

#### Fluoride conclusions

The conclusion from these laboratory and clinical studies is that there is a major anticaries effect for high caries individuals if a "therapeutic level" of fluoride as a background at around 0.1 ppm F in saliva can be achieved day and night. Any additional fluoride delivery, such as twice daily brushing with a fluoride toothpaste, will be a bonus. A sustained-release device that functions to provide the same protection as the glass device referred to above should be the target, only in a more acceptable form to the patients. Such a device would overcome compliance problems and could be targeted with success to high caries risk individuals. It may not eliminate all caries, but would lead to dramatic reductions, and in concert with antibacterial treatments could indeed eliminate caries in these individuals.

### Chlorhexidine

Caries is a transmissible infection. Although the principle of using antibacterial therapy to reduce the caries challenge has been around for a long time, it is not considered in general dental practice. Fluoride is the primary anticaries entity that is considered, and drilling and filling is the primary method to "fix" caries. Unfortunately, placing a restoration only removes the bacteria from that particular cavity and does nothing to lower the bacterial levels in the remainder of the mouth [14]. If the caries balance concept is applied (Figure 1), then for high caries risk individuals antibacterial therapy as well as fluoride therapy is needed to swing the balance to a no-caries status. Fluoride therapy alone is insufficient to deal with high caries challenge. If it were sufficient, then there would be very little caries to consider, and presentations such as the present one would be unnecessary. Therefore, we must consider currently available antibacterial therapy against dental caries organisms.

Chlorhexidine has been shown in numerous studies to reduce the levels of *mutans streptococci *(MS) in the plaque biofilm in human mouths [15,16]. It is much less effective at reducing the levels of lactobacilli (LB) in human mouths. The effect of Chlorhexidine on other acid-producing bacteria has been little studied. We need an antibacterial that also attacks effectively the lactobacilli species that are strongly related to caries progression. Although Chlorhexidine is effective at killing MS and LB in the laboratory, it is much less effective in the mouth when these organisms are in a biofilm. This is especially the case for LB. It is not known whether this is a substantivity issue, or a diffusion related phenomenon, or whether MS and LB deeper in the biofilm cannot be effectively reached for some other reason. What is known is that chlorhexidine reduces MS but does not usually eliminate it except with intensive, high-concentration, repeated applications [17]. Other common antibacterials such as cetyl pyridinium chloride and phenol are not effective against the cariogenic organisms in the biofilm in the mouth.

Although numerous studies have investigated the reduction of MS and/or LB in the mouth as a result of Chlorhexidine therapy, most have not continued to a clinical caries end point (see review by Anderson, 2003) [15]. A recently completed caries clinical trial investigated whether changing caries risk status reduced the caries outcome [14]. High caries risk adults (1–8 frank cavities at baseline) were treated with Chlorhexidine rinse and fluoride rinse (0.05% NaF) over a three-year period. The control group was given "conventional care" with no Chlorhexidine. Bacterial and fluoride levels in saliva were assayed at six-month intervals in both groups. Chlorhexidine (0.12% as gluconate) was self-applied as a mouth rinse by the subjects in the study once a day either for two weeks every three months or one week every month. The Chlorhexidine therapy significantly reduced the MS levels during the 12 months that the restorative work was done, but did not significantly reduce the LB levels. The combined Chlorhexidine and fluoride rinse therapy significantly reduced the mean overall caries risk and significantly reduced the mean new caries in the test group. For some subjects the therapy was very successful, whereas in others it was not.

Compliance with the use of rinses, especially one that tastes as bad as Chlorhexidine, is a major problem to be solved. The above-mentioned study conclusively proved that reducing the bacterial levels, and thereby tipping the caries balance favorably, led to reduced caries risk, and most importantly, a reduction in new caries.

Although Chlorhexidine varnish has been marketed, a recent clinical trial did not show any caries reducing beneficial effect [18], having only an initial effect on reducing the levels of MS. In light of the above results for multiple applications of Chlorhexidine rinse, it is obvious that the bacteria levels can be reduced, but not eliminated. Therefore, ongoing antibacterial treatments are needed to bring down the levels each time the bacteria attempt to re-establish their pathological levels.

#### Chlorhexidine conclusions

Intervention with multiple applications over periods of months of 0.12% Chlorhexidine gluconate can significantly reduce MS levels, and in combination with fluoride therapy can markedly lower the risk for future caries. However, the Chlorhexidine is much less effective against LB in the mouth. Current Chlorhexidine products require patient compliance with a rinse that tastes bad, has the potential to stain, and has to be applied numerous times to be effective. Work is needed to develop a delivery system that avoids the need for patient compliance, provides ongoing multiple doses as needed over months, and has a carrier that provides access and killing power to the LB in the biofilm. An improved antibacterial that is effective against both MS and LB and has a daily-dosage mechanism would be optimal.

### Xylitol

Xylitol is a five carbon sugar alcohol that looks and tastes like sucrose, and is therefore a good "sugar substitute." Further benefits are that it is not metabolized by the cariogenic bacteria and that it has antibacterial properties (see review Lynch and Milgrom, 2003, available at )[19]. Unlike Chlorhexidine mouthrinse, which cannot be used by young children and infants because they cannot effectively rinse and spit, and they do not tolerate the taste, Xylitol tastes like sucrose, is sweet, and is non-cariogenic. It is ideal for infants and young children, provided a suitable delivery vehicle can be developed. Xylitol has great potential because not only does it provide a substitute for the fermentable carbohydrates on the pathological side of the caries balance (Figure 1), but it also appears to function as an antibacterial on the protective side of the balance.

Several studies have shown the clinical benefits of xylitol in markedly inhibiting the transfer of bacteria from mother to child. In a six-year clinical trial Soderling and coworkers [20,21] showed that mothers who chewed xylitol gum during the first two years of the children's lives had highly significant reductions in colonization of MS, and, very importantly, had dramatically less dental decay five years later. This study and others[19] clearly show the benefits of xylitol chewing gum in caries reduction by inhibiting bacterial transfer and colonization. A recent study in 5-year-olds further showed that chewing xylitol gum over a six-month period actually reduced levels of MS in the mouth [22]. Plaque scores were also reduced.

Chewing gum and mints are now available in the USA from a variety of suppliers, and xylitol wipes that could be used in a baby's mouth have recently become available. The obvious question is this: What delivery vehicles can be used for young children and infants to provide useful dosage levels?

#### Xylitol Conclusions

Xylitol is a sucrose substitute that appears to be not only non-cariogenic but also has antibacterial properties. It inhibits transfer of cariogenic bacteria from person to person and reduces bacterial recolonization over time.

Several questions remain regarding xylitol. These are:

1) What is the optimum dose per day to have an anticaries effect?

2) Are there any undesirable side effects at the optimum dosage level?

3) What delivery vehicles other than chewing gum are suitable, and for what ages?

4) Are xylitol mints, which have a delivery period of a few seconds, as effective as xylitol gum, which has a prolonged delivery period?

5) Are there any long-term negative effects of xylitol if it is delivered daily to infants, children, or adults?

6) How can xylitol products be successfully marketed in the US when therapeutic claims are not made?

## Conclusion

There is now considerable information available on dosage levels for fluoride if delivered in a slow-release, long-term delivery form. What remains is to develop an acceptable delivery system that elevates the baseline salivary fluoride levels into a truly therapeutic window.

Chlorhexidine could be much more effectively delivered as a successful antibacterial against MS, but a better antibacterial is needed, especially one that is effective against LB in the biofilm. Antibacterial therapy in conjunction with fluoride therapy can be very effective at reducing or eliminating caries in high risk individuals. However, the antibacterial therapy must be delivered in a form that overcomes lack of compliance, since high caries risk individuals are likely to be poor compliers.

Xylitol shows great promise not only as a sugar substitute, but also as an antibacterial that can be used in infants and for the inhibition of transfer from person to person. New delivery vehicles are needed to effectively deliver xylitol at therapeutically effective levels over time.

## Competing interests

The author(s) declare that they have no competing interests.
